# Synthetic biology and microbioreactor platforms for programmable production of biologics at the point-of-care

**DOI:** 10.1038/ncomms12211

**Published:** 2016-07-29

**Authors:** Pablo Perez-Pinera, Ningren Han, Sara Cleto, Jicong Cao, Oliver Purcell, Kartik A. Shah, Kevin Lee, Rajeev Ram, Timothy K. Lu

**Affiliations:** 1 Synthetic Biology Group, Department of Biological Engineering and Electrical Engineering & Computer Science, Massachusetts Institute of Technology, Cambridge, Massachusetts 02142, USA; 2 Research Laboratory of Electronics, Massachusetts Institute of Technology, Cambridge, Massachusetts 02142, USA; 3 The David H. Koch Institute for Integrative Cancer Research, Massachusetts Institute of Technology, Cambridge, Massachusetts 02139, USA; 4 Pharyx Inc., Woburn, Massachusetts 01801, USA; 5 The Broad Institute of MIT and Harvard, Cambridge, Massachusetts 02412, USA

## Abstract

Current biopharmaceutical manufacturing systems are not compatible with portable or distributed production of biologics, as they typically require the development of single biologic-producing cell lines followed by their cultivation at very large scales. Therefore, it remains challenging to treat patients in short time frames, especially in remote locations with limited infrastructure. To overcome these barriers, we developed a platform using genetically engineered *Pichia pastoris* strains designed to secrete multiple proteins on programmable cues in an integrated, benchtop, millilitre-scale microfluidic device. We use this platform for rapid and switchable production of two biologics from a single yeast strain as specified by the operator. Our results demonstrate selectable and near-single-dose production of these biologics in <24 h with limited infrastructure requirements. We envision that combining this system with analytical, purification and polishing technologies could lead to a small-scale, portable and fully integrated personal biomanufacturing platform that could advance disease treatment at point-of-care.

## Abstract (web summary)

Current biopharmaceutical manufacturing platforms use single biologic-producing cell lines cultured at large scales. Here the authors develop a small-scale, portable biomanufacturing platform to produce single dose IFNa2b and rHGH from a single engineered strain of *P. pastoris*.

## Graphical abstract

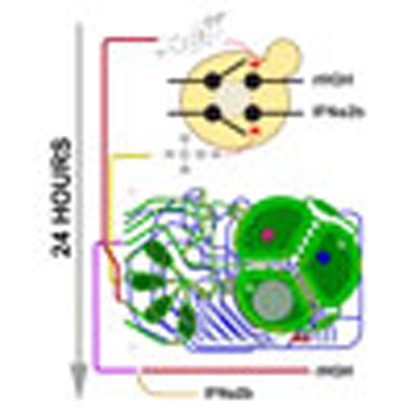

Biopharmaceutical production is one of the largest contributors to escalating expenditures in healthcare, in particular due to the length, complexity and high costs associated with product discovery and development as well as the cost of construction and scale-up of biomanufacturing^1^,^2^,^3^. In addition, biologics treatment of patients in remote parts of the world, battlefields, emergency situations or under-developed regions with poor infrastructure presents formidable logistical challenges that greatly impact drug availability at the point-of-care^4^,^5^,^6^,^7^,^8^,^9^.

Currently, manufacturing of biologic drugs in the biopharmaceutical industry relies heavily on large-scale fermentation batches that are frequently monitored offline, to ensure a robust process and consistent quality of product^4^,^8^,^10^. However, as personalized medicines, single-use technologies and the desire for global and decentralized access to biologics are becoming increasingly important, there is a growing need for rapid, flexible, scalable and portable biomanufacturing systems that can be monitored/controlled online for affordable, safe and consistent production of biologics^11^.

To address this demand for personalized biomanufacturing technologies, we developed a new platform for flexible and portable production of biologic therapeutics at the point-of-care in short time frames and with limited system requirements. The basic components of this platform are as follows: (1) a biologics expression system engineered to secrete multiple therapeutic proteins in response to programmable cues and (2) an integrated, small overall footprint, millilitre-scale perfusion microfluidic platform capable of supporting rapid and flexible biomanufacturing process control that enables portable operations in mobile units, such as vehicles (Fig. 1 and Supplementary Movie 1). We demonstrate that this system produces near-single-dose levels of recombinant human growth hormone (rHGH) and interferon-α2b (IFNα2b) in <24 h.

## Results

### Host selection

Recombinant biologics for therapeutic use in humans can be produced using a variety of host organisms, including bacteria, yeast, plants, insect cells and mammalian cells^12^. The specific host used can have an impact on yields, need for viral inactivation, downstream purification requirements, as well as final product formulation. Mammalian Chinese Hamster Ovary (CHO) cells are the most commonly used host for producing Food and Drug Administration (FDA)-approved biologics^9^, but they have complex media requirements and their storage requires cryopreservation. Therefore, the long time needed to go from inoculation of biologic-producing CHO cells to release of a drug product, which meets established quality standards and an FDA-approved safety profile, is incompatible with a rapid production system. Yeast are attractive alternatives to CHO cells, as they have simple media requirements, grow quickly to high densities and can be stored as lyophilized material^12^,^13^. *Pichia pastoris* (also known as *Komagataella phaffi*) is becoming increasingly popular for biologic production, as it (1) can grow to very high densities on simple and inexpensive carbon sources; (2) has a strong yet tightly controlled alcohol oxidase 1 (AOX1) promoter, which can be induced by methanol for high level protein production (up to 10 g l^−1^) and is effectively repressed by glycerol or glucose; (3) is capable of human-like posttranslational modifications, including glycosylation^14^,^15^,^16^; and (4) secretes heterologous proteins into the extracellular space very efficiently with minimal host protein contamination, thus requiring relatively simple downstream purification systems^17^,^18^.

To date, more than 500 different proteins, including simple peptides, enzymes, hormones, monoclonal antibodies and FDA-approved therapeutics have been expressed in *P. pastoris*^1^,^4^,^5^. Thus, as a proof-of-concept of flexible biologics manufacturing using a single host, we developed strains of *P. pastoris* to support the independently selectable production of two different therapeutics. The use of individual strains that support production of multiple biologics provides significant advantages over the use of multiple strains that each produce single biologics. First, leveraging strains that produce multiple biologics for rapid production of dosage-level biologics enables the re-use of accumulated biomass from the outgrowth period. This approach dramatically improves production speed by avoiding the need to regrow different strains into production-level biomass. Second, for production of multi-component products, the FDA requires approval of multiple manufacturing lines that each produce individual components and thus a single multiplexed expression platform could offer a potential regulatory advantage.

The AOX1 promoter (P_AOX1_) is useful for producing one protein on-demand. Only a few alternative inducible promoters have been characterized in *P. pastoris*, including the CUP1, G1 or FLD1 promoters. However, it remains unclear whether these promoters support high levels of expression, while remaining orthogonal to P_AOX1_ (refs 19, 20, 21). Thus, to enable selectable bioproduction, we sought to develop new inducible promoters that are orthogonal to and can surpass P_AOX1_ in promoter strength.

### Transformation platform

Current transformation methods for *P. pastoris* rely on genomic integration of small linearized plasmids through homologous recombination, followed by antibiotic selection and screening for high-copy-number integrants^22^. Although these approaches are adequate for simple genetic manipulations, such as introducing small expression cassettes, they have several limitations for more sophisticated synthetic biology applications. In addition, multiple random integration events are undesirable when attempting to compare expression levels between different genetic constructs.

**Figure 2:**
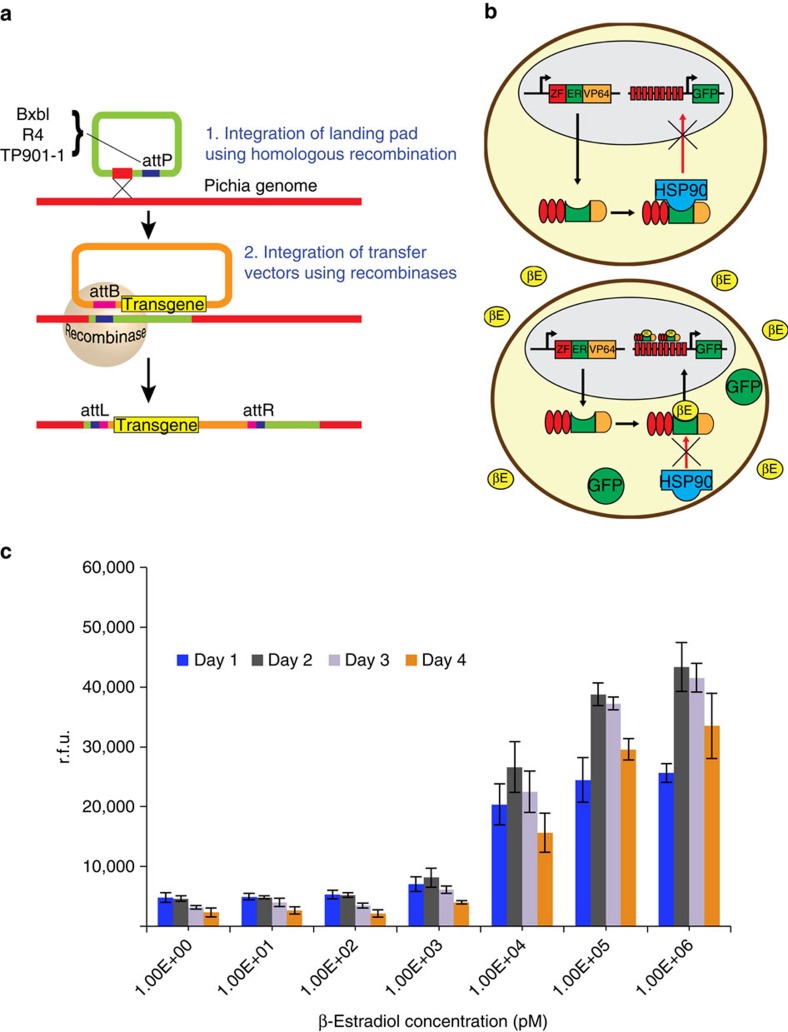
Development of artificial promoter systems for high-level transgene expression in *P. pastoris.* (**a**) Schematic representation of the landing-pad-based integration system used in these studies. We first generated a parental strain containing landing pads based on *attB* sites for the recombinases BxbI, R4 and TP-901.1. This strain can be efficiently transformed with a transfer vector containing the desired gene circuit and the corresponding *attP* site together with a plasmid encoding the corresponding recombinase. (**b**) Schematic representation of the β-estradiol-inducible system used for in this study. This system uses a ZF DNA-binding domain fused to the β-estradiol-binding domain of the human oestrogen receptor, which is coupled to a transcriptional activation domain. This synthetic ZF-TF is sequestered in the cytoplasm by HSP90. Addition of β-estradiol displaces HSP90 and permits translocation of the ZF-TF into the nucleus, where it activates expression of genes regulated by a minimal promoter placed downstream of multiple ZF-binding sites. (**c**) Dose–response and time course of GFP expression using the β-estradiol-inducible system, where the *S. cerevisiae* TEF1 promoter was used to express the ZF-TF, and a minimal GAP promoter preceded by nine binding sites of ZF43-8 was used for inducible expression of GFP. β-Estradiol (0.1–1 μM) is necessary to achieve full activation of this system at 48 h. Error bars represent s.e.m. (*n*=3).

To accelerate the rapid design-test-and-optimize cycle for creating new promoters, we first sought to overcome the rate-limiting step of plasmid transformation and genomic integration of synthetic constructs into *P. pastoris*. We created a recombinase-based system for the single-copy integration of plasmids at defined loci that is suitable even for large DNA constructs (Fig. 2a). We first generated a parent *P. pastoris* strain containing *attB* sites for the recombinases BxbI, R4 and TP-901 (ref. 23). This was accomplished by traditional integration of a construct containing regions of homology to the Trp2 locus in the *P. pastoris* genome (Supplementary Tables 1 and 2, Integration Site 1), *attB* sites and a *KanR* selection cassette. Integration at the Trp2 locus was validated using PCR and a copy number of 1 was verified using quantitative PCR (qPCR; Supplementary Fig. 1). We then co-transformed a plasmid for the transient expression of BxbI, R4 or TP901, together with a transfer plasmid containing *attP* sites for the corresponding recombinase and engineered genetic constructs of interest. This method typically generated ∼50–300 transformants per reaction for DNA constructs ranging from ∼7.8 to 13.6 kb (Supplementary Fig. 2).

### Optimization of β-estradiol-inducible expression systems

Using this integration strategy, we implemented an inducible transcriptional system consisting of a constitutively expressed zinc-finger (ZF) DNA-binding domain^24^ fused with the β-estradiol binding domain of the human oestrogen receptor, which is coupled with a transcriptional activation domain^25^. At steady state, this synthetic ZF transcription factor (ZF-TF) is sequestered in the cytoplasm by HSP90. Addition of β-estradiol displaces HSP90 and permits translocation of the ZF-TF into the nucleus, where it activates expression of genes regulated by a minimal promoter placed downstream of multiple ZF-binding sites (Fig. 2b). This system offers a highly flexible architecture that can be tuned by modifying different parameters, including the following: (1) affinity of the DNA-binding domain to DNA; (2) strength of the transcriptional activation domain; (3) number of binding sites for the ZF; (4) promoter driving expression of the ZF; (5) minimal promoter driving expression of the output; (6) dose of inducer; and (7) integration site.

Initial tuning of expression levels was performed with green fluorescent protein (GFP) as the output quantified using flow cytometry. The ZF DNA-binding domain used in these experiments was ZF43-8 (ref. 24). We fused the ZF DNA-binding domain and β-estradiol-binding domain of the human oestrogen receptor to the VP64 transcriptional activation domain, which has been previously shown to mediate higher levels of expression in mammalian cells than other domains, such as p65 or VP16 (ref. 26). The *Saccharomyces cerevisiae* TEF1 promoter was used to express the ZF-TF and a minimal GAP promoter preceded by nine binding sites for ZF43-8 was used to drive the inducible expression of GFP. At 24 h, dose–response curves showed that maximum expression of GFP could be attained with only 0.01 μM β-estradiol (Fig. 2c). However, at 48 h, 0.1–1 μM β-estradiol was necessary to fully saturate this system.

**Figure 3:**
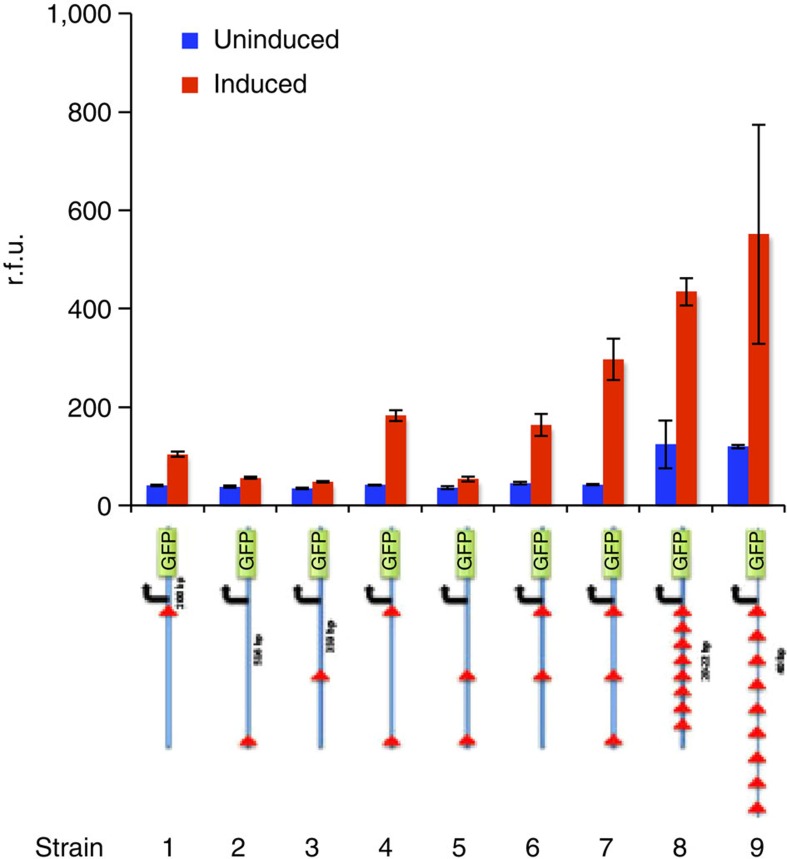
Optimization of ZF-binding sites (triangles) in the minimal promoter for estradiol-inducible expression. Different combinations of three ZF-binding sites were placed ∼200, ∼350 or ∼500 bp from the ATG, as well as binding sites spaced ∼20 or ∼40 bp from each other. The results show that more ZF-binding sites yield higher levels of GFP expression, whereas changing the spacing between binding sites did not significantly improve levels of expression. Error bars represent s.e.m. (*n*=3).

We further optimized gene expression by modulating the number and placement of ZF-binding sites within the artificial promoter, which have been shown to be important parameters for maximizing the expression of regulated genes^27^. We used a ppTEF1 promoter to express the ZF-TF, which targeted a minimal CYC promoter preceded by single ZF-binding sites placed ∼200, ∼350 or ∼500 upstream of the ATG start codon, as well as combinations of two or three ZF-binding sites. We also tested one promoter with nine binding sites spaced ∼20 bp and one promoter with nine ZF-binding sites spaced ∼40 bp from each other (with the closest ZF-binding site located 200 bp upstream of the ATG codon of the output gene). We found that promoters with nine binding sites expressed GFP at higher levels than promoters with three binding sites when induced, whereas increasing the spacing between binding sites did not significantly improve levels of expression (Fig. 3).

Chromosomal context is an important factor to consider when expressing heterologous genes, because promoter interference, chromatin structure or other epigenetic modifications can have a negative impact on gene activation^28^,^29^. Thus, we tested GFP expression from cassettes integrated at nine different chromosomal loci. These loci correspond to regions targeted in prior reports^22^,^30^ or to intergenic regions, to avoid directly disrupting native coding sequences (Supplementary Tables 1 and 2). We did not observe statistically significant differences in the maximally induced levels of gene expression across nine loci (Supplementary Fig. 3).

The performance of the ZF-inducible expression system can be further affected by two additional parameters: the strength of the promoter driving expression of the ZF-TF and the basal activity of the minimal promoter, which contains ZF-binding sites, that controls expression of the output. The promoter driving ZF-TF expression is constitutive and determines how much ZF-TF accumulates in the cytoplasm. Excessive ZF-TF levels can potentially surpass the capacity of HSP90 to sequester the ZF-TF in the cytoplasm and, as a result, the ZF-TF may spontaneously translocate into the nucleus and activate expression in the absence of inducer, thus increasing undesired background. To minimize background expression levels, the minimal promoter containing ZF-binding sites should activate gene expression only when the inducer is present.

**Figure 4:**
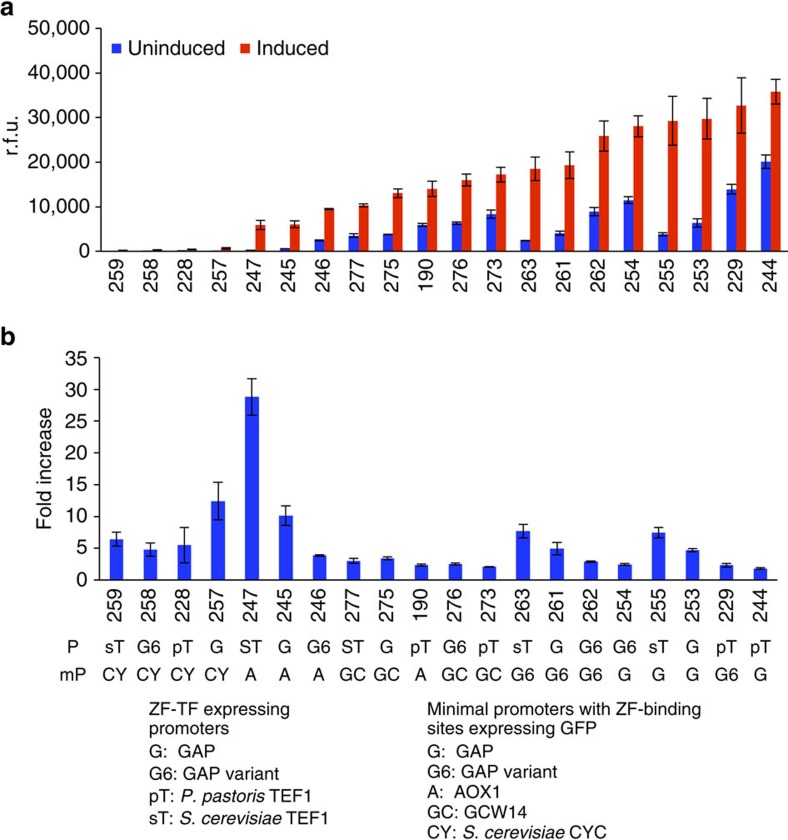
Promoter optimization for high-level estradiol-inducible expression. (**a**) Four different promoters were used to express the ZF-TF, including the GAP promoter, a variant GAP promoter and the TEF1 promoter from *P. pastoris*, as well as the *S. cerevisiae* TEF1 promoter. We used five different minimal promoters containing nine ZF-binding sites located upstream of the ATG codon that are targeted by the ZF-TF to control GFP expression. The different combinations exhibited a wide range of background expression and maximal activation after 24 h of induction with β-estradiol in BMGY. Error bars represent s.e.m. (*n*=3). (**b**) Fold increase in levels of GFP expression after induction with estradiol. Error bars represent s.e.m. (*n*=3).

To fine-tune the expression of our output signal, we expressed the ZF-TF from several constitutive promoters previously used in *P. pastoris*, as well as variations of the GAP promoter that we constructed by introducing ∼50 bp insertions throughout its DNA sequence to modify its activity (Supplementary Fig. 4). We built combinations of these ZF-TF-expressing constitutive promoters with minimal promoters derived from those that regulate AOX1, GAP, a GAP6 variant, GCW14 and *S. cerevisiae* CYC1 (Fig. 4). We measured GFP expression via flow cytometry with and without induction with β-estradiol. The different combinations exhibited a wide range of background expression levels and maximal activation ratios (Fig. 4a). Interestingly, some of the combinations consistently supported maximum expression levels higher than those reached by GFP expressed directly from the well-characterized P_AOX1_ under maximal induction (Supplementary Fig. 5). Overall, combinations that exhibited higher maximum expression levels also had greater background levels in the OFF state.

**Figure 5:**
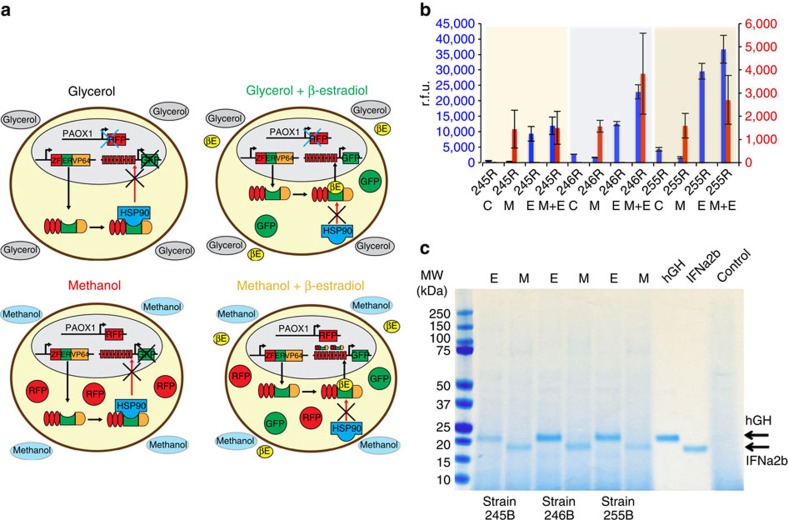
Selectable biologics production from *P. pastoris* strains with both an estradiol-inducible circuit and a methanol-inducible circuit. (**a**) Three strains containing circuit architectures with acceptable ON/OFF ratios were selected (245, 246 and 255) and added an RFP expression cassette controlled by the methanol-inducible AOX1 promoter (245R, 246R and 255R). (**b**) These strains expressed GFP when induced with β-estradiol in BMGY, expressed RFP when induced with BMMY and expressed both GFP and RFP when induced with β-estradiol in BMMY for 24 h. Error bars represent s.e.m. (*n*=3). (**c**) Strains 245B, 246B and 255B were generated by replacing GFP and RFP in strains 245R, 246R and 255R with rHGH and IFNα2b. Protein gel electrophoresis and Coomassie staining of supernatants from shake flasks demonstrated high-level expression of the desired biologic drug on stimulation for 24 h in BMGY for β-estradiol or BMMY for methanol.

We selected three architectures that had ON/OFF ratios in excess of ∼4-fold and/or high maximal expression activities for further engineering (architectures 245, 246 and 255; Fig. 4b). We then added an expression cassette to produce red fluorescent protein (RFP) using the AOX1 promoter to these vectors and generated strains 245R, 246R and 255R, respectively (Fig. 5a). As expected, these strains expressed GFP when induced with β-estradiol in buffered glycerol-complex medium (BMGY), expressed RFP when induced with buffered methanol-complex medium (BMMY) and expressed both GFP and RFP when induced with β-estradiol in BMMY (Fig. 5b).

Using the synthetic expression cassettes that were optimized with intracellular fluorescent reporters 245R, 246R and 255R (Fig. 5b), we then built a proof-of-concept system to controllably produce two biologic drugs, rHGH and IFNα2b. This resulted in three strains capable of selectable expression of two different biologics: strain 245B (GAP promoter expressing ZF-TF, AOX1 minimal promoter with ZF-binding sites expressing rHGH and AOX1 promoter expressing IFNα2b), strain 246B (GAP6 promoter expressing ZF-TF, AOX1 minimal promoter with ZF-binding sites expressing rHGH and AOX1 promoter expressing IFNα2b) and strain 255B (scTEF1 promoter expressing ZF-TF, GAP minimal promoter with ZF-binding sites expressing rHGH and AOX1 promoter expressing IFNα2b). Strain 245B, 246B and 255B were capable of selectable production of high-dose rHGH or IFNα2b in shake flasks within 24 h of induction (Fig. 5c).

Interestingly, the level of protein secretion was similar from the AOX1 promoter or the β-estradiol-inducible system when rHGH expression was induced after 48 h of outgrowth (Supplementary Fig. 6). One difference between the systems is that β-estradiol can be used to induce protein secretion without outgrowth, while using glycerol as a carbon source, thereby allowing growth and production to occur simultaneously. Methanol induction, however, requires biomass accumulation (typically in glycerol) before the induction phase^22^. Without prior biomass accumulation, protein production from AOX1 was significantly lower than production from a β-estradiol-inducible promoter (Supplementary Fig. 7). As the daily dose of rHGH needed to treat patients is higher than the dose of IFNα2b, we engineered *P. pastoris* strains for expression of rHGH on β-estradiol stimulation and expression of IFNα2b on methanol stimulation. To maximize protein production, we further tested different media additives and determined that formulations containing the antifoams L81, P2000 and AF204 enhanced rHGH secretion levels from strain 255, where GFP was replaced with rHGH (Supplementary Fig. 8).

### Continuous and selectable protein production in microbioreactors

Sequential manufacturing of multiple biological therapeutics from individual strains requires rapid changes to their environment in high-density cell cultures. Furthermore, manufacturing at the volume scale of individual doses (for example, millilitres) suits requirements for point-of-care applications. For example, a production yield of 120 μg ml^−1^ for IFNα2b in yeast^31^ could result in multiple doses when produced at the millilitre scale, as a common formulation of IFNα2b (Intron-A, db00105) is 11.6 μg (ref. 32). These two requirements can be complementary as the large surface-area-to-volume ratio afforded by miniature systems facilitates rapid media changeover. To demonstrate this, we developed a protocol specifically tailored for programmable protein production with our engineered strains in an integrated, millilitre-scale table-top microbioreactor that can be operated continuously for point-of-care use in personal biomanufacturing, even with limited resources. By combining our engineered dual-biologics strain with the operational flexibility of the microbioreactor device, we extended the traditional biomanufacturing approach, where a single biologic is produced per process, into one that enables sequential or controllable expression of multiple different biologics.

**Figure 6:**
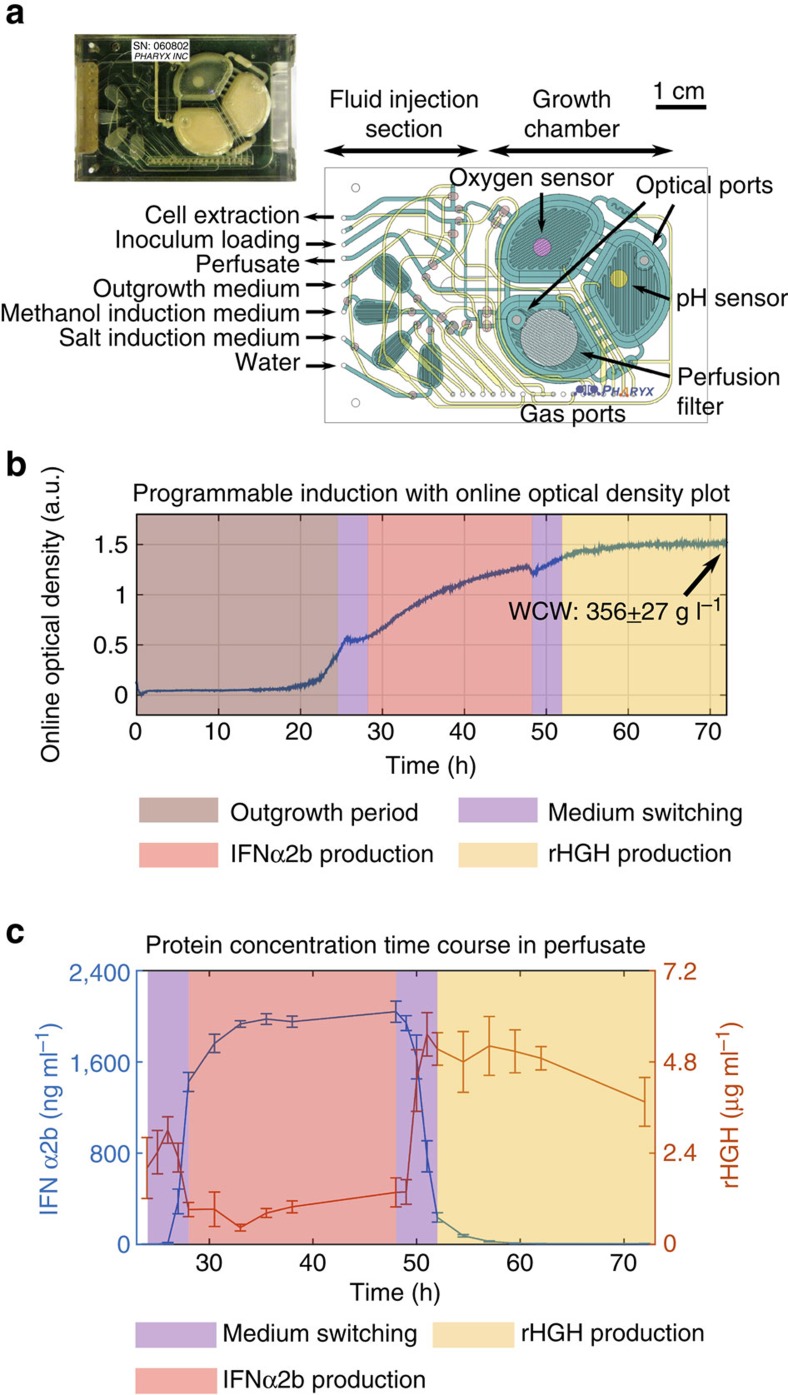
Programmable protein production in a millilitre-scale table-top microbioreactor. (**a**) The principal component of the microbioreactor is a polycarbonate-PDMS membrane-polycarbonate sandwiched chip with active microfluidic circuits that are equipped for pneumatic routing of reagents, precise peristaltic injection, growth chamber mixing and fluid extraction. (**b**) Three-day continuous cultivation experiments for selectable production of two biologics were performed. The different operational phases are colour coded in the online OD plot for one experiment. The microbioreactor enabled high-density cell cultures up to a wet-cell weight (WCW) of 356±27 g l^−1^. (**c**) Samples were collected at the indicated time points and protein production was measured using ELISA, which demonstrated near single-dose drug production levels in fewer than 24 h. Error bars represent s.e.m. (*n*=4).

The principal component of the microbioreactor is a polycarbonate-Polydimethylsiloxane (PDMS) membrane-polycarbonate sandwiched chip with active microfluidic circuits outfitted pneumatically for routing of reagents, precise peristaltic injection, growth chamber mixing and fluid extraction (Fig. 6a)^33^. We used an injection volume of 700∼900 nl per injection for precise control of fluid addition/extraction in the 1-ml volume growth chamber. A 0.8-μm pore size perfusion filter (polyethersulfone) with a 1-cm diameter was incorporated underneath the growth chamber, to allow for fluid flow-through while maintaining all of the cells inside the growth chamber and enabling the switching of induction media (Fig. 6a). The ratio of the filter surface area to bioreactor volume was 0.758—a factor of 3 higher than high-performance, bench-scale perfusion bioreactors previously reported^34^.

To demonstrate the paradigm of personalized, single-dose and programmable biomanufacturing, we performed 3-day continuous cultivation experiments for selectable production of two biologics at near-single-dose levels in <24 h. The dual-biologics-producing *P. pastoris* strain 255B was inoculated from a single colony and grown in BMGY, first in batch and then in perfusion mode, with a perfusion rate of 0.5 ml h^−1^. At 24 h, the outgrowth media was switched to our custom methanol media with perfusion rates of 1 ml h^−1^ for 4 h for rapid changeover of the chemical environment, followed by IFNα2b collection at a perfusion rate of 0.5 ml h^−1^ for 20 h. The 1 ml h^−1^ changeover rate was chosen to ensure that >98% of the preceding medium had been flushed out after this 4-h medium changeover period according to our rate equation model for microbioreactor operation (see ‘Microbioreactor flow modelling' in Methods). After the 48-h time point, we switched the custom methanol media with the custom β-estradiol-containing media at a perfusion rate of 1 ml h^−1^ for 4 h, followed by a collection phase for rHGH lasting 20 h at a perfusion rate of 0.5 ml h^−1^. A summary of the microbioreactor control operation is provided in Table 1. The different operational phases are illustrated with the online optical density (OD) plot in Fig. 6b. This experimental procedure resulted in 10 ml of perfusate for each protein production period lasting 20 h each.

During the cultivation, cells inside the growth chamber were rapidly circulated and mixed by peristalsis. The fermentation temperature was controlled at 30±0.1 °C. The online OD was recorded through an optical path length of ∼250 μm by a 630-nm light-emitting diode, where the optical path length is chosen to maximize the linear response range compatible with our fabrication process. The dissolved oxygen level of the culture was monitored online and controlled by dynamically changing the gas feed line between air and oxygen to match the dissolved oxygen set point, which was set to 100% air saturation in the experiment. The online pH data were also recorded during the fermentation process. The real-time sensor data for the microbioreactor experiments presented here can be found in the Supplementary Fig. 9. A video showing the peristaltic mixing and optical sensing in the microbioreactor during the culture is provided in Supplementary Movie 2. We used four different input ports for the injection of BMGY outgrowth media, custom-made methanol media for IFNα2b production, β-estradiol-containing media for rHGH production and water for evaporation compensation. Perfusate was collected through the fluid channel downstream of the perfusion filter for protein characterization. For portable storage operation where lyophilized material may be used, the lyophilized material can be re-suspended in the reconstitution media before inoculation. This can serve as the seed inoculum and be injected into the growth chamber during the inoculation process. The cells could be revived inside the growth chamber subsequently without the need of additional steps. If the reconstitution media differs from the outgrowth media, a perfusion media changeover to the outgrowth media can be performed after the revival process.

To understand induction dynamics and protein secretion levels, we collected samples every hour during the 4-h media switching periods. In addition, during the 20-h production periods, we first collected four samples every 2.5 h and then one sample after the last 10 h. A standard enzyme-linked immunosorbent assay (ELISA) was carried out to quantify protein production. As shown in Fig. 6c, production profiles for the two biologics corresponded to the different induction media toggled by the microbioreactor. Four parallel microbioreactor experiments were carried out at the same time with identical protocols. Induction with methanol resulted in the rapid secretion of IFNα2b, which reached maximal productivity after only 3 h and remained constant during the production period, and then rapidly decreased after methanol was removed from the media. Similarly, rHGH secretion was induced via media changeover to the custom β-estradiol-containing media, thus demonstrating multi-product expression control in our integrated platform.

A summary of cumulative protein production and measurements of wet cell weight at the end of the experiment averaged across the four microbioreactors is shown in Table 2. The total average production of IFNα2b was 19.73 μg per reactor, which exceeds the 11.6 μg dose in Intron-A, whereas the total average production of rHGH was 43.7 μg per reactor (a common starting weight-based formulation of rHGH (Nutropin) is 0.006 mg kg^−1^ per day^35^). Therefore, even without extensive bioprocess optimization, this system is capable of producing IFNα2b in excess of the daily dose needed in adults and matching the daily dose rHGH required to treat infants. Importantly, the cultivation conditions in the perfusion microbioreactor provided continuous nutrient supplies and high oxygen transfer rates^33^ that led to the highest reported cell culture density achieved in any microfluidic platform, measured as an average wet cell weight of 356±27 g l^−1^ (ref. 36). In addition to the microbioreactor run shown in Fig. 6, three additional microbioreactor runs operating the same protocol were carried out. The additional results are shown in Supplementary Fig. 10.

## Discussion

The choice of therapeutic molecules and the regulatory gene circuits used in a multiplexed biomanufacturing platform is important. For example, our current expression systems can achieve ∼110-fold and ∼4-fold ON:OFF ratios for IFNα2b and rHGH, respectively. However, concerns about background production of biologic A during induction of biologic B and vice versa could make downstream purification processes and the regulatory approval process more challenging. Using recombinase-based switches to invert protein-expressing DNA cassettes^37^,^38^ or additional repressors could reduce leakage beyond the transcriptional systems used here. For example, biologics-expressing cassettes could be surrounded by recombinase-recognition sites and initially encoded in inactive positions; expression of a recombinase could invert a targeted cassette and allow an upstream promoter to transcribe the correct messenger RNA. In addition, translational repressors or RNA interference could be used to further knock down undesirable expression levels in uninduced conditions. Combinatorial assembly of large numbers of genetic circuits followed by high-throughput screening for ones with enhanced ON:OFF ratios could further lower background expression. The integration of purification platforms with our biomanufacturing system could also help reduce background levels of biologics in uninduced states. To circumvent the limited number of inducible systems available for controlling biologics expression, future work could integrate more advanced gene circuits, such as multiplexers that enable a restricted set of *n* inputs to control the expression of 2^*n*^ outputs. Additional inducible systems that leverage orthogonal chemical inputs or non-chemical inputs, such as light, could further help with the scalability of this system and, in the latter case, reduce logistics requirements.

Producing multi-component products such as vaccines could also circumvent the issue of undesirable background production, as multiple products could be expressed from a single strain. Furthermore, synthetic biology could help tailor a vaccine for specific populations, as different antigens are likely to be optimal in providing immunity depending on geographical location or timing. With artificial regulation over the expression of different antigens, we could control the ultimate formulation of multi-component vaccines on demand for optimal prophylaxis and mitigate concerns about background expression. These vaccines could be customized to specific outbreaks or local conditions to enhance their applicability. Currently, the production of such multi-component vaccines can require multiple manufacturing lines, each with its own FDA approval. A multiplexed expression platform for multi-component vaccines could conceivably reduce the regulatory burden for such products. Similarly, the controlled expression of multi-component biologic products could also benefit from this approach and help mitigate concerns over leaky background expression.

By combining genetic and hardware engineering, we have demonstrated a proof-of-concept, integrated and highly flexible platform for the programmable production of two biopharmaceuticals, IFNα2b and rHGH. Future work includes scaling up the number of products that can be controllably manufactured, selecting specific drug candidates that are compatible with each other, enhancing production levels while reducing background expression levels and integrating this system with purification, polishing and analytics^39^. We envision that such technologies will enable the creation of small-scale, portable, fully integrated and closed biomanufacturing systems that can advance the treatment of human diseases at the point-of-care.

## Methods

Different media formulations namely BMGY, yeast extract peptone dextrose (YPD), BMMY, custom-made methanol medium and β-estradiol-containing salt medium were used in these experiments. BMGY medium contained 10 g l^−1^ (1% (w/v)) yeast extract (VWR catalogue #90004-092), 20 g l^−1^ (2% (w/v)) peptone (VWR catalogue #90000-264), 100 mM potassium phosphate monobasic (VWR catalog #MK710012), 100 mM potassium phosphate dibasic (VWR catalog #97061-588), 4 × 10^−5^% biotin (Life Technologies #B1595), 13.4 g l^−1^ (1.34% (w/v)) Yeast Nitrogen Base (Sunrise Science catalogue #1501-500) and 2% glycerol (VWR catalogue #AA36646-K7). YPD contained 1% yeast extract, 2% peptone and 2% dextrose (VWR catalogue # BDH0230). BMMY contained 1% yeast extract, 2% peptone, 100 mM potassium phosphate monobasic, 100 mM potassium phosphate dibasic, 4 × 10^−5^% biotin and 10 ml l^−1^ (1% (v/v)) methanol (VWR catalogue #VWRCBDH20864.4). The custom-made methanol medium contained 1.34% Yeast Nitrogen Base, 0.79 g l^−1^ casaminoacids (Fisher #BP1424-500), 2% methanol (VWR catalogue #VWRCBDH20864.4) and 0.1% antifoam 204 (Sigma-Aldrich catalogue #A8311-50ML). The β-estradiol-containing salt medium contained 30 μM β-estradiol (Sigma-Aldrich catalogue #E4389-100MG), 18.2 g l^−1^ K_2_SO_4_ (VWR catalogue #97062-578), 7.28 g l^−1^ MgSO_4_ (Sigma-Aldrich catalogue #M7506-500G), 4.3 g l^−1^ KOH (Fisher #P250-1), 0.08 g l^−1^ CaSO_4_ 2H_2_O (Sigma Aldrich catalogue #C3771-500G), 13 ml l^−1^ 85% orthophosphoric acid (VWR catalogue #E582-50ML), 1.47 g l^−1^ sodium citrate (Fisher #S279-500), 0.1% antifoam 204 and pH adjusted to 5.5 with ammonium hydroxide (Sigma-Aldrich catalogue #318612-500ML). All individual reagents were prepared as stock solutions and mixed immediately before the experiments. BMGY, custom-made methanol medium and β-estradiol-containing salt medium were used in the experiment for initial outgrowth, IFNα2b production and rHGH production, respectively.

### Plasmid construction

The multiple constructs used in these experiments were built using conventional restriction enzyme cloning and/or Gibson assembly using the vector pPICZ A (Invitrogen #V190-20) as the backbone. All plasmids used in this manuscript are described in Supplementary Table 3 and have been deposited in the Addgene plasmid repository.

### Strains

The wild-type *Pichia pastoris* strain NRRL Y- 11430, ATCC 76273 (reclassified as *Komagataella phaffii*) was used in this study.

### Electroporation

Competent cells were prepared by first growing one single colony of *P. pastoris* in 5 ml YPD at 30 °C overnight. Fifty microlitres of the resulting culture were inoculated in 100 ml of YPD and grown at 30 °C overnight again to an OD_600_ ∼1.3–1.5. The cells were then centrifuged at 1,500 *g* for 5 min at 4 °C and resuspended with 40 ml of ice-cold sterile water, centrifuged at 1,500 *g* for 5 min at 4 °C and resuspended with 20 ml of ice-cold sterile water, centrifuged at 1,500 *g* for 5 min at 4 °C and resuspended in 20 ml of ice-cold 1 M sorbitol, and centrifuged at 1,500 *g* for 5 min at 4 °C and resuspended in 0.5 ml of ice-cold 1 M sorbitol. For transformation of the landing pad plasmids, 80 μl of competent cells were mixed with 5–20 μg of linearized DNA and transferred to an ice-cold 0.2 cm electroporation cuvette for 5 min. For recombinase-based transformations, 10 μg of circular transfer vector and 10 μg of circular recombinase expression vector were combined, added to 80 μl of competent cells and incubated for 5 min in an ice-cold 0.2 cm electroporation cuvette. Pulse parameters were 1,500 V, 200 Ω and 25 μF. Immediately after pulsing, 1 ml of ice-cold 1 M sorbitol was added to the cuvette and the cuvette content was transferred to a sterile culture tube containing 2 × YPD. The culture tubes were incubated for 2 h at 30 °C with shaking and 50–100 μl of the culture was spread on Yeast Extract Peptone Dextrose Sorbitol (YPDS) plates (1% yeast extract, 2% peptone, 1 M sorbitol, 1% dextrose and 2% agar) with the appropriate selection antibiotic (zeocin 100 μg ml^−1^; G418 100 μg ml^−1^).

### Cell induction and flow cytometry

One single colony was grown in 1 ml of BMGY in a 12-ml culture tube at 30 °C in a shaking incubator (250–300 r.p.m.) overnight. The cells were centrifuged at 500 *g* for 5 min at room temperature and washed twice with PBS. After the second wash, the cells were resuspended in induction medium consisting of BMMY or BMGY with β-estradiol. After 24 h, the cultures were centrifuged at 500 *g* for 5 min at room temperature, washed with 1 ml of PBS twice, resuspended in 1 ml of PBS and used for flow cytometry with a LSR Fortessa cell analyser.

### ELISA assay

The concentration of hGH and IFN-α2b in each of the samples was determined by ELISA assay. Solid-phase 96-well ELISA plates were used, specifically designed for the quantification of hGH (Quantikine ELISA, R&D Systems) and IFN-α (Verikine Human IFN-alpha ELISA Kit, PBL Assay Science). The products were provided with the proper standard stock solutions or powder for each of the assays. For hGH, the standard curve was calculated using concentrations ranging between 3,200 and 25 ρg ml^−1^, in twofold serial dilutions. For IFNα2b, the extended range standard curve was used, with an additional large concentration point: the standard concentration varied between 156 and 10,000 ρg ml^−1^, in twofold serial dilutions. For both assays, negative controls were also included. Before the protein quantification, the samples were diluted in the appropriate buffer so that the concentrations determined would fall within the assay and equipment limits.

The optical density values obtained for each of the assays were plotted using a four-parameter fit for the standard curve. Each sample was measured twice and the results represent the average and s.d. of three biological replicates.

### Quantitative PCR

Genomic DNA from different strains was isolated using the YeaStar Genomic DNA kit (Zymo) and the resulting preps were diluted down to a concentration of 5 ng μl^−1^. qPCR mixtures were prepared using the LightCycler 480 SYBR Green Master mix (Roche) with 10 ng genomic DNA from each strain was used with 400 nM of each primer per assay in a total reaction volume of 20 μl. Reactions were performed in LightCycler 480 96-well reaction plates in triplicate with a standard curve for each gene generated from serial ten-fold dilutions of 2 ng of plasmid DNA containing the gene-of-interest. The amplification conditions were as follows: 95 °C for 10 min followed by 45 cycles of 95 °C for 10 s, 56 °C for 15 s and 72 °C for 20 s. The amplification period was followed by a melting curve analysis with a temperature gradient of 0.1 °C s^−1^ from 65 °C to 95 °C. An amplicon from the single-copy *GAPDH* or *ACT1* genes was used for normalization. Using the published genome size of 9.4 Mbp, we expected 98,000 copies of the genome to be present in 1 ng haploid *P. pastoris* genomic DNA. Absolute copy number for the gene-of-interest in each strain was calculated using the mean Ct value and the corresponding standard curve.

The sequence of the primers used were as follows: hGH (5′-GGGCAGATCTTCAAGCAGAC-3′, 5′-CTCGACCTTGTCCATGTCCT-3′); IFN (5′-TTCCCACAAGAGGAATTTGG-3′, 5′-AGGCTGCTGAGGAATCTTTG-3′); GAPDH (5′-TGGGTTACACTGAAGATGCC-3′, 5′-CGTTGTCGTACCAAGAGATCAG-3′); ACT1 (5′-TGGTATCGTTTTGGACTCTGG-3′, 5′-AGCGTGTGGTAAGGAGAAAC-3′); and landing pad (5′-TGTCTTCGTGGTTTGTCTGG-3′, 5′-TCTTGTAGTTGCCGTGTCG-3′).

### Microbioreactor

Parts for the microbioreactor experiment were purchased from Pharyx Inc. A single bioreactor control hub (Pharyx, #MBS-004) with an overall footprint of 31 cm (w) × 34 cm (d) × 36 cm (h) was used to control four independent microbioreactor units (Pharyx, #MCM-001) for the fermentation experiment. Each microbioreactor unit interfaces with a single-use disposable microfluidic chip made of a sandwiched polycarbonate-PDMS membrane-polycarbonate structure (Pharyx, #CCPST-1) to carry out peristaltic control and online sensing. The design, fabrication and system configuration for the microbioreactor have been described previously^33^,^40^. To provide the rapid medium changeover required for this study, a perfusion filter (Pall Corp., Supor 800, #60109) was incorporated into the cultivation chamber^41^ to allow medium flow-through, while maintaining cells inside the growth chamber.

### Microbioreactor flow modelling

To account for the microbioreactor media concentration dynamics during the changeover period, a simple model based on fluid concentration rate equation under equal inflow and outflow rate was constructed. For simplicity, cellular consumption on media material was not taken into account in this model. Assuming that *S* (*t*) represents a particular medium concentration in the growth chamber, *S*_in_ (*t*) represents the concentration of this medium in the input flow, *F* represents the input flow rate and *V* represents the growth chamber volume, then the rate equation for the medium concentration in the growth chamber can be modelled as


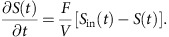


For the situation of introducing a completely new medium into the growth chamber to replace an old medium similar to our programmable biologics production experiment, assuming *S*_in_ (*t*) is constant during the time of changeover and *t*=0 being the start of changeover event, the new medium concentration can be simply solved analytically as


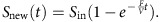


Meanwhile, the concentration of the old medium due to flush out follows as


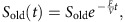


assuming *S*_old_ is the concentration of the old medium before the changeover event. Therefore, a flow rate of 1 ml h^−1^ for the changeover period of 4 h would result in a medium replacement percentage of ∼1−*e*^−4^≈98.2%. In contrast, if the changeover rate stays as 0.5 ml h^−1^ similar to the case for the production period, the medium replacement percentage would be ∼1−*e*^−2^≈86.5%, where there would still be a substantial amount of the previous medium leftover after the changeover event. Such physical modelling of the microbioreactor operation provides a simple yet effective design guideline to complement the operational flexibility of our manufacturing platform.

### Microbioreactor experiment

The microbioreactor chips were γ-irradiated and sealed as part of the standard pre-inoculation sterile protocol. The medium bottles and feed lines were autoclaved separately. The initial inoculum was loaded from a single colony from a YPD plate stored at 4 °C at 0 h. The fermentation parameter plot for the online OD, dissolved oxygen, pH and temperature for one experiment is shown in Supplementary Fig. 9. As described in the manuscript, the fermentation temperature was controlled at 30±0.1 °C throughout the entire experiment. The dissolved oxygen level of the microbioreactor was controlled by dynamically changing the gas feed line between air and oxygen to match the dissolved oxygen set point, which was set to 100% air saturation in the experiment. As the cells grow and the overall oxygen consumption rate increases, the gas controller gradually increases the oxygen content in the gas feed line to maintain the dissolved oxygen set point. Once the cell oxygen consumption rate overpasses the oxygen transfer rate by pure oxygen supply, the dissolved oxygen drops below the set point and the supply gas remains at 100% oxygen. The online OD is monitored through light scattering across an optical path length of ∼250 μm inside the growth chamber with a 630-nm light-emitting diode. The linear response range for this OD sensor is around 0∼0.7 online OD unit. Above ∼0.7 online OD unit, the sensor reading no longer increases linearly with the cell density. The online pH data are also recorded during the fermentation process. The pH sensor is rated for pH values of 5.5∼8.5. During the third day, the pH reading falls below 5.5 and therefore may not accurately represent the actual pH of the culture environment. Over the course of the study, perfusate samples were collected downstream of the perfusion filter and were stored at 4 °C before processing with ELISA assay.

### Additional microbioreactor runs

In addition to the microbioreactor run presented in the body manuscript, three additional runs of the microbioreactor experiments were performed over the course of 5 months. Each run is marked by the time of the experiment and consists of two independent microbioreactors operating the same protocol in parallel. The protein concentration time course plot is shown in Supplementary Fig. 10 and the cumulative protein production quantity is summarized in Supplementary Table 4. Overall, the same switching behaviour was observed across all runs with some run-to-run protein production variations observed, especially for the December 2014 Run, where rHGH production was much higher than the rest. These run-to-run variations are probably caused by our experimentation with the inner surface coating and γ-irradiation protocol of the microbioreactor chip. After the microbioreactor parts were fabricated and bonded, an inner surface coating protocol with polyethylene glycol (PEG)-silane treatment was carried out, followed by a chip nitrogen purge and bagging procedure, and γ-irradiation sterilization. Some experimentation on this protocol in terms of PEG-silane treatment time and chip nitrogen purge procedure in search for the optimal coating condition was carried across the early microbioreactor runs. For all runs, the chips used in the same run were fabricated with exactly the same protocol and therefore do not cause much variation within each microbioreactor run. As we standardized our chip fabrication protocol during this 5-month time period, our run-to-run variations reduced substantially (the March 2015 Run and the main body run that happened in June 2015 had much less production variations compared with the rest). Nonetheless, the result from the March 2015 Run suggests the potential of high production yield comparable to industrial level for our manufacturing platform with suitable manufacturing standardization and bioprocess optimization.

### Data availability

The data that support the findings of this study are available from the corresponding author upon request. The plasmids used in this manuscript have been deposited in Addgene. Addgene accession numbers can be found in Supplementary Table 3.

## Additional information

**How to cite this article:** Perez-Pinera, P. *et al*. Synthetic biology and microbioreactor platforms for programmable production of biologics at the point-of-care. *Nat. Commun.* 7:12211 doi: 10.1038/ncomms12211 (2016).

## Supplementary Material

Supplementary Information Supplementary Figures 1-10, Supplementary Tables 1-4

Supplementary Movie 1 The portable microbioreactor system operating in the context of an ambulance.

Supplementary Movie 2 Mixing and optical sensing cycles for a microbioreactor with highdensity yeast culture.

## Figures and Tables

**Figure 1:**
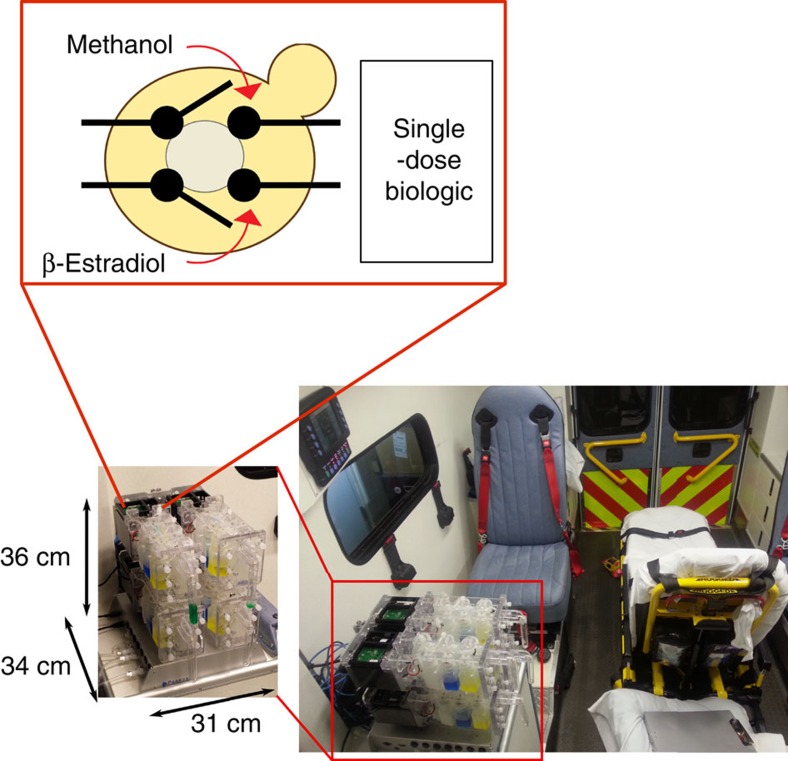
Point-of-care biomanufacturing by integrating genetically engineered strains and portable microbioreactors for programmable biologics expression. *P. pastoris* strains genetically engineered to contain independently controllable inducible genetic cassettes were used within portable perfusion microbioreactors to achieve high-density and programmable expression of two biologic drugs. Here we show the microbioreactor system in the context of an ambulance.

**Table 1:** Microbioreactor operation.

Time	Medium	Flow rate	Purpose
0 h	BMGY	0	Outgrowth
14–24 h	BMGY	0.5 ml h^−1^	Outgrowth
24–28 h	Methanol medium	1.0 ml h^−1^	Rapid medium switchover
28–48 h	Methanol medium	0.5 ml h^−1^	IFNα2b production
48–52 h	β-Estradiol medium	1.0 ml h^−1^	Rapid medium switchover
52–72 h	β-Estradiol medium	0.5 ml h^−1^	rHGH production

BMGY, buffered glycerol-complex medium; IFNα2b, interferon-α2b; rHGH, recombinant human growth hormone.

Summary table including the microbioreactor operation timeline, inflow medium, flow rate and purpose for different operation phases in Fig. 6b.

**Table 2:** Microbioreactor production.

IFNα2b	rHGH	IFNα2b	rHGH	Wet cell weight
Production (20 h)	Production (20 h)	Leakage (20 h)	Leakage (20 h)	
19.73±0.72 μg	43.7±6.3 μg	0.177±0.032 μg	10.8±2.9 μg	356±27 g l^−1^

IFNα2b, interferon-α2b; rHGH, recombinant human growth hormone.

Production summary for IFNα2b and rHGH, and wet cell weight measurement at the end of the experiment. All data are averaged across four independent microbioreactors running the same protocol in parallel. Values represent mean and s.e.m. (*n*=4).
