# Supplementary material for: Synthetic biology and microbioreactor platforms for programmable production of biologics at the point-of-care
Source: Nat Commun. 2016 Jul 29;7:12211. doi: 10.1038/ncomms12211 (PMC4974573; doi:10.1038/ncomms12211)
Supplement: Supplementary Information — Supplementary Figures 1-10, Supplementary Tables 1-4 [file ncomms12211-s1.pdf]

A

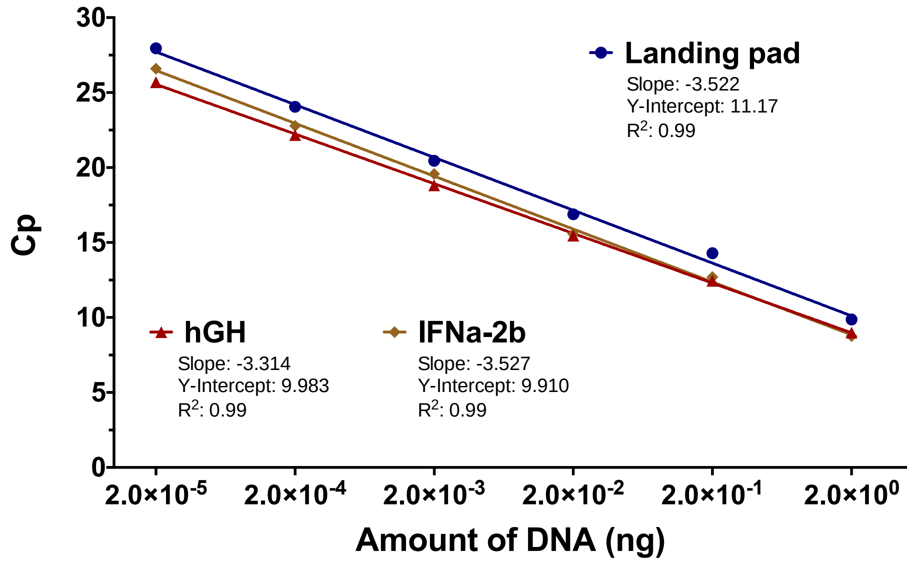

B

Strain	Cp	Absolute copy number
Landing pad (PP74)	18.32	1.24
hGH (255B)	17.17	0.96
IFN (255B)	16.84	1.17

Supplementary Figure 1: Results of qPCR to measure copy number of the landing pad as well as of IFNa2b- and rHGH-expression constructs integrated in the genome of the PP74 and PP255 strains used in these studies. **A**: Standard curves generated from qPCR amplification of plasmids carrying the landing pad, hGH or IFNa-2b genes. **B**: Crossing point (Cp) values generated form qPCR amplification of 10 ng isolated genomic DNA for the above-mentioned genes. Cp values were used to determine absolute copy numbers. The results demonstrate that the copy number was ~ 1 in all strains (n=3).

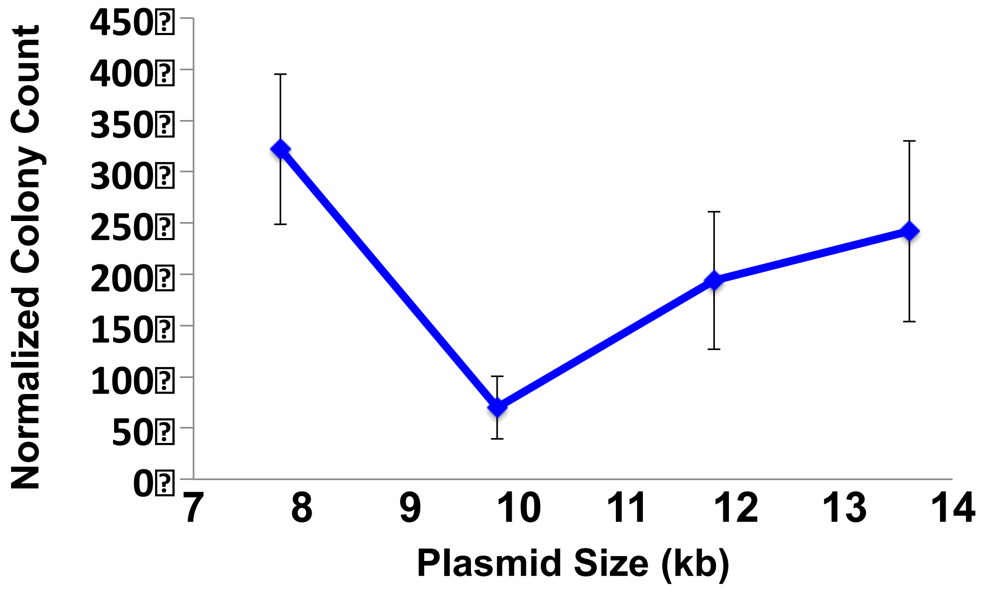

Supplementary Figure 2: The recombinase platform that we developed was used to integrate plasmids of different sizes in the landing pad containing *attB* sites for the recombinases BxbI, R4, and TP-901 in the genome of *P. pastoris* (Integration Site 1). The number of colonies obtained 3 days after transformation by electroporation (as described in materials and methods) was approximately constant regardless of plasmid size. Equal moles of plasmid were transformed for each size of plasmid. Error bars represent s.e.m. (n=5)

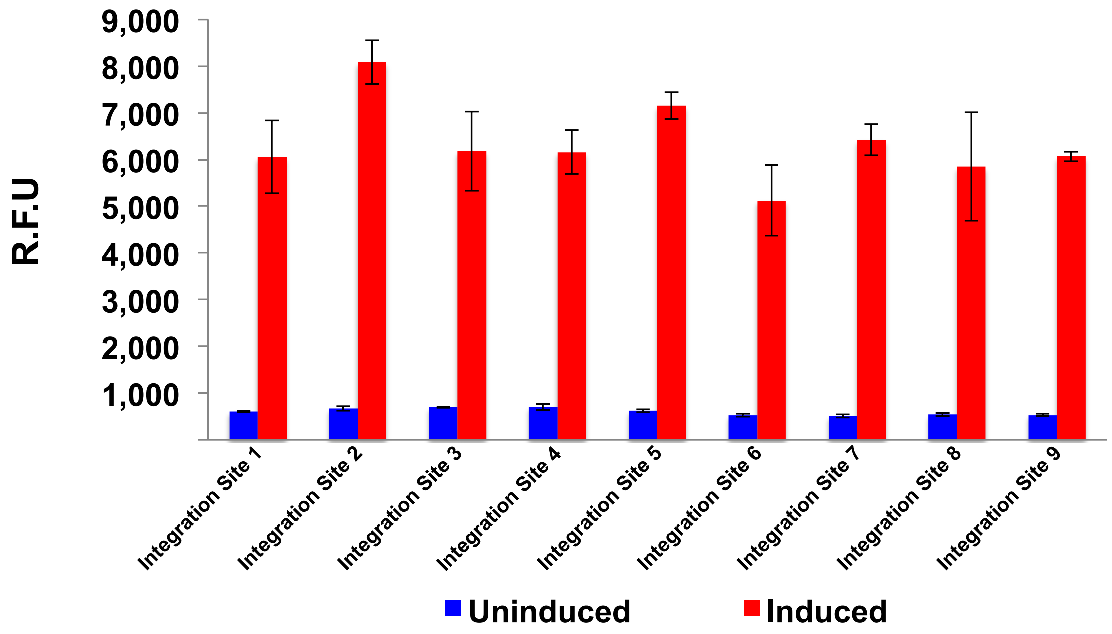

Supplementary Figure 3: Testing of genomic loci for high-level gene expression from the β-estradiol-inducible system. The landing pad was integrated at 9 different loci in the genome of *P. pastoris*. The recombinase BxbI was used to integrate a β-estradiol-inducible system that controls expression of GFP. The different strains were induced with 1 μM β-estradiol and fluorescence was measured using flow-cytometry. Single factor ANOVA determined that there are no statistically significant differences between the groups. Error bars represent s.e.m. (n=3).

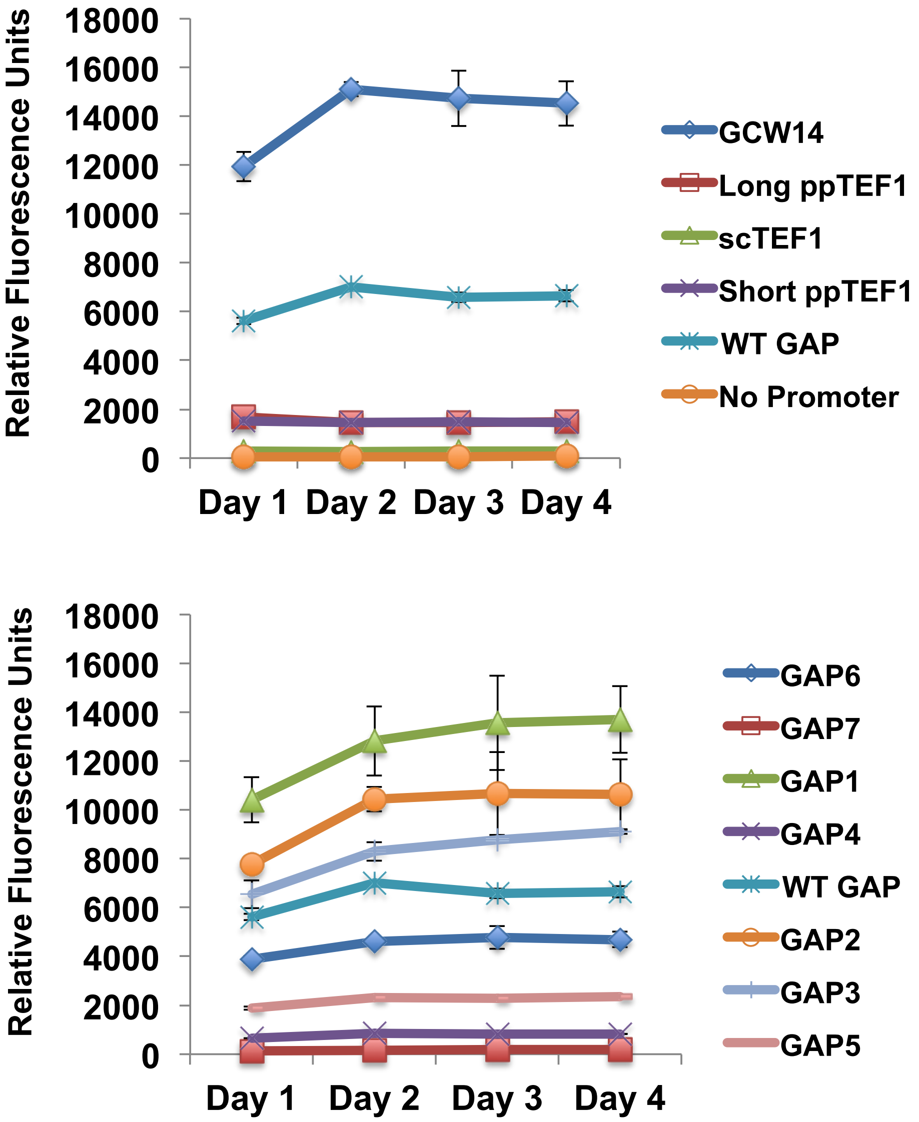

Supplementary Figure 4: Engineered *P. pastoris* strains that express GFP constitutively from the promoters GCW14, *S. cerevisiae* TEF1 (scTEF1), long and short versions of *P. pastoris* TEF1 (ppTEF1) and GAP. Furthermore, we introduced 2 TetO sites at different positions within the GAP promoter to create GAP promoter variants (GAP1-7), which resulted in a broad range of constitutive GFP expression. Error bars represent s.e.m. (n=2).

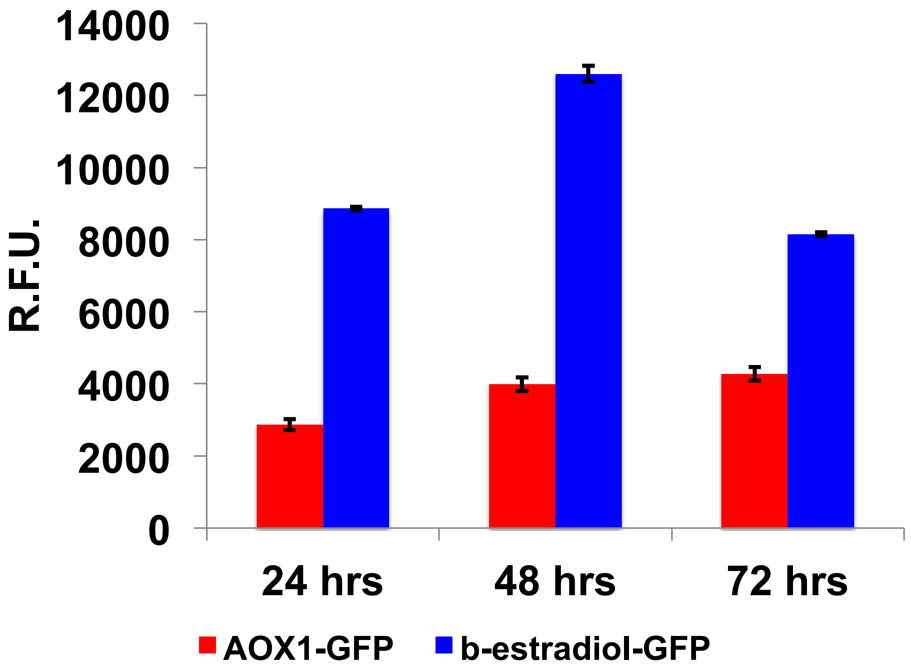

Supplementary Figure 5: *P. pastoris* strains were engineered to express GFP under the methanol-inducible AOX1 promoter (strain AOX1-GFP) or under the synthetic β-estradiol-inducible promoter (strain 255). Expression of GFP was induced after 24 hours of outgrowth with β-estradiol in BMGY or with BMMY and measured at 24, 48 and 72 hours using flow cytometry. The β-estradiol-inducible system supports levels of expression superior that those observed using the methanol-inducible system. Error bars represent s.e.m. (n=3).

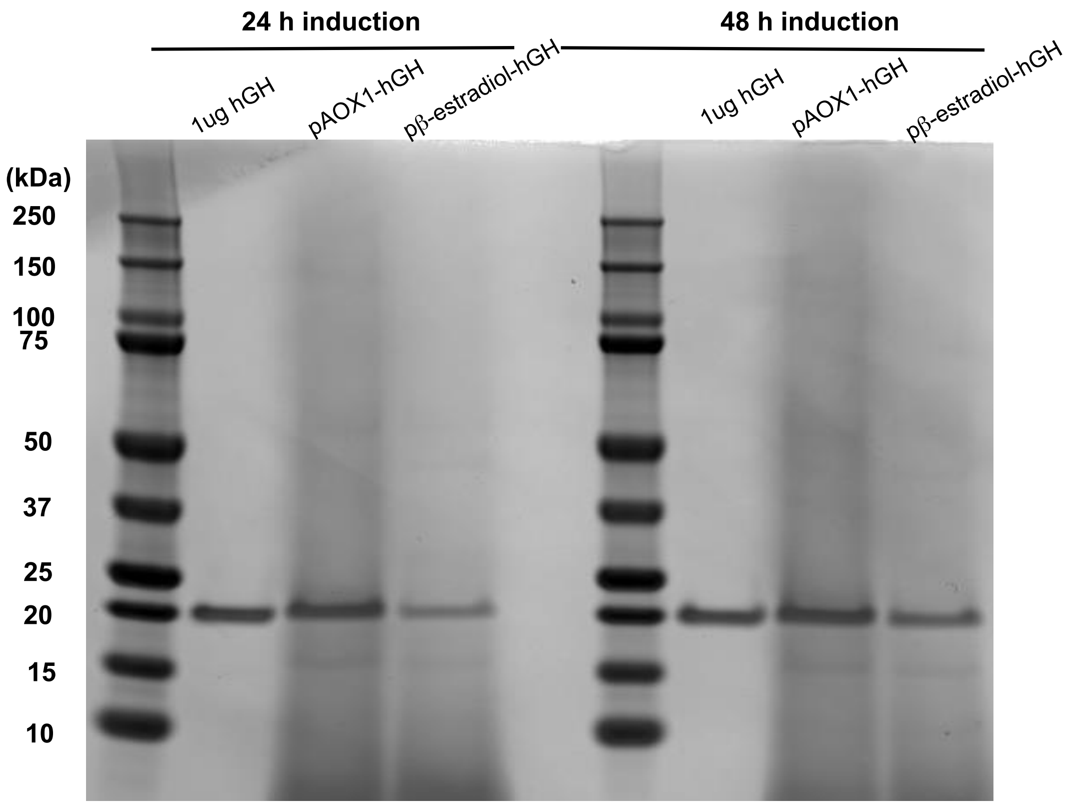

Supplementary Figure 6: Side-by-side comparison of rHGH production induced by methanol in BMMY when rHGH expression is regulated by the AOX1 promoter (strain AOX1-rHGH) or when expression of rHGH is induced by β-estradiol (strain 255, where GFP was replaced with rHGH) in BMGY with a ZF-based inducible system. The cells were induced after 48 hours of outgrowth and culture supernatant was collected at 24 h and 48 h post-induction and analyzed by PAGE and Coomassie staining. The results demonstrate that in this growth condition, both systems are comparable.

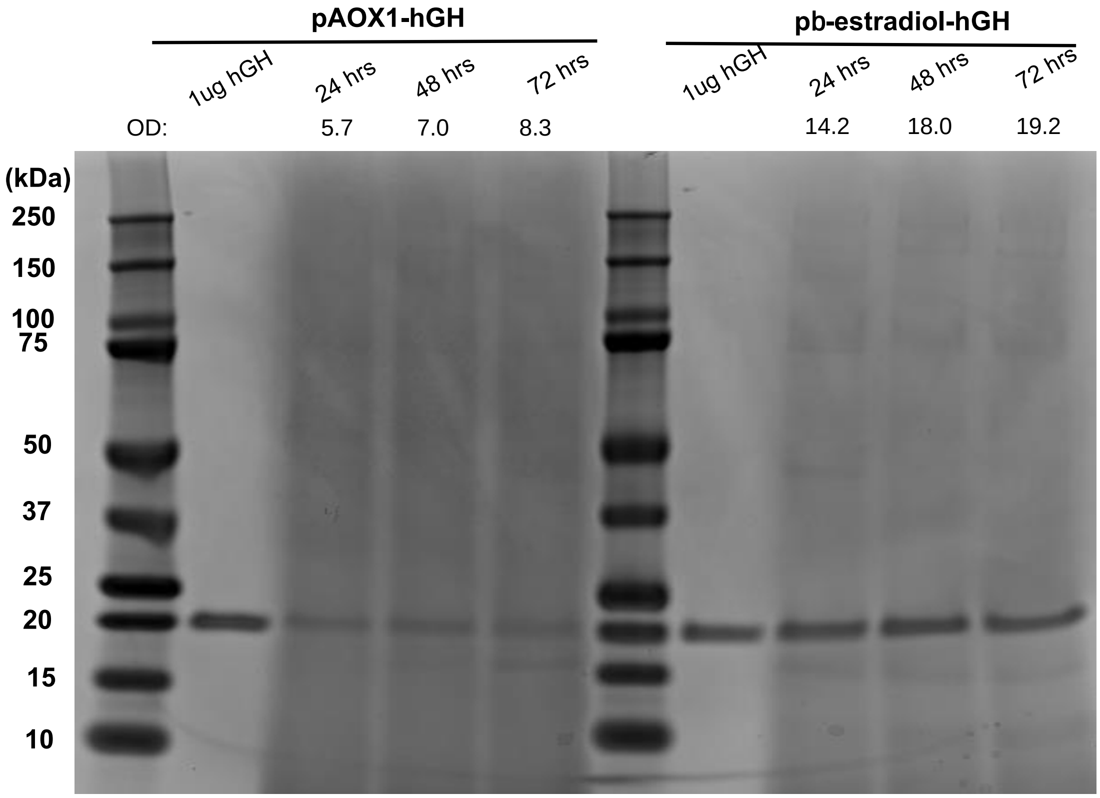

Supplementary Figure 7: Side-by-side comparison of rHGH production from a strain with the AOX1 promoter (strain AOX1-rHGH) induced by methanol versus a strain with the β-estradiol-inducible system (strain 255, where GFP was replaced with rHGH) induced with β-estradiol in BMGY at low OD with minimal outgrowth. The culture supernatant was analyzed using PAGE and Coomassie staining. Since β-estradiol induction is compatible with glycerol as a carbon source, both growth and protein production can occur simultaneously. However, when methanol is used as an inducer and carbon source, growth is reduced and less biomass is available to support high-level protein secretion.

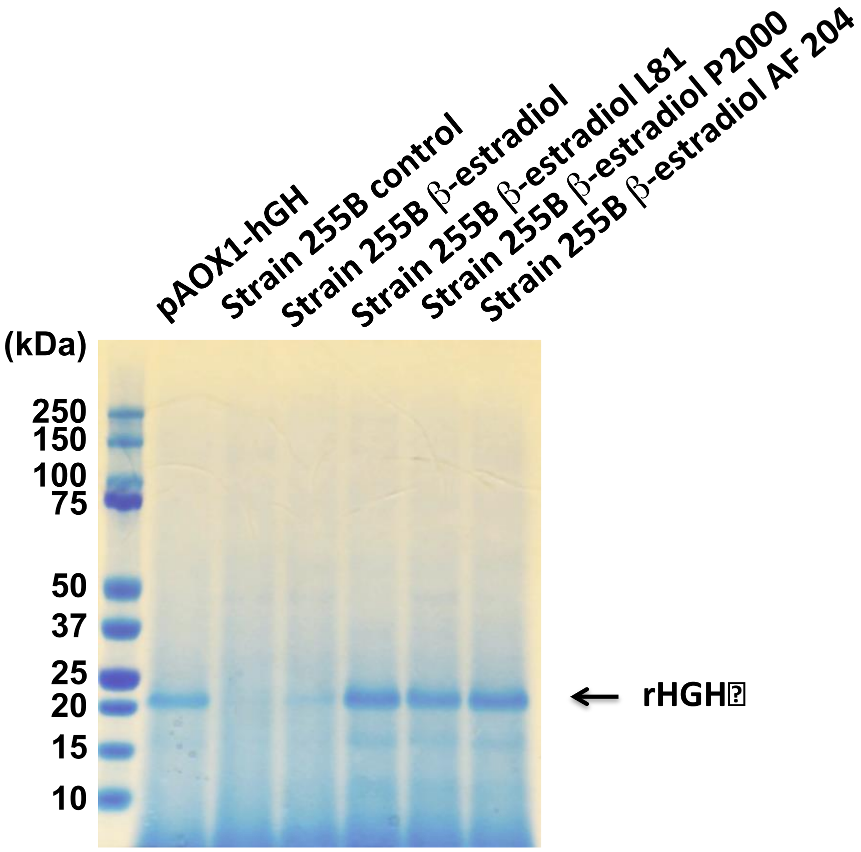

Supplementary Figure 8: Different media formulations were tested to identify conditions that facilitate not only high levels of expression but also high levels of secretion. Strain 255B was grown for 24 hours and then induced for 24 hours with β-estradiol in BMGY with or without antifoams. Overall, addition of the antifoams L81, P2000, and AF204 to the induction media enhanced rHGH secretion levels.

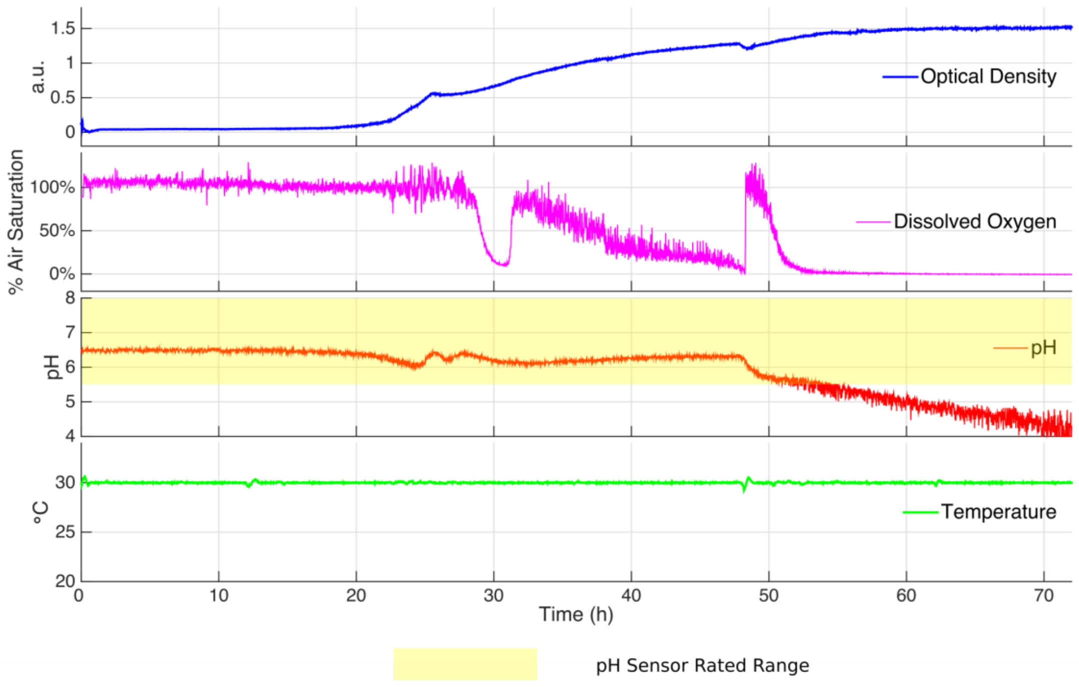

Supplementary Figure 9: The real-time online optical density, dissolved oxygen, pH and temperature measurement plots for one microbioreactor experiment. Note that the online pH sensor used in our study is rated for pH values of 5.5 ~ 8.5, which is highlighted in yellow in the plot.

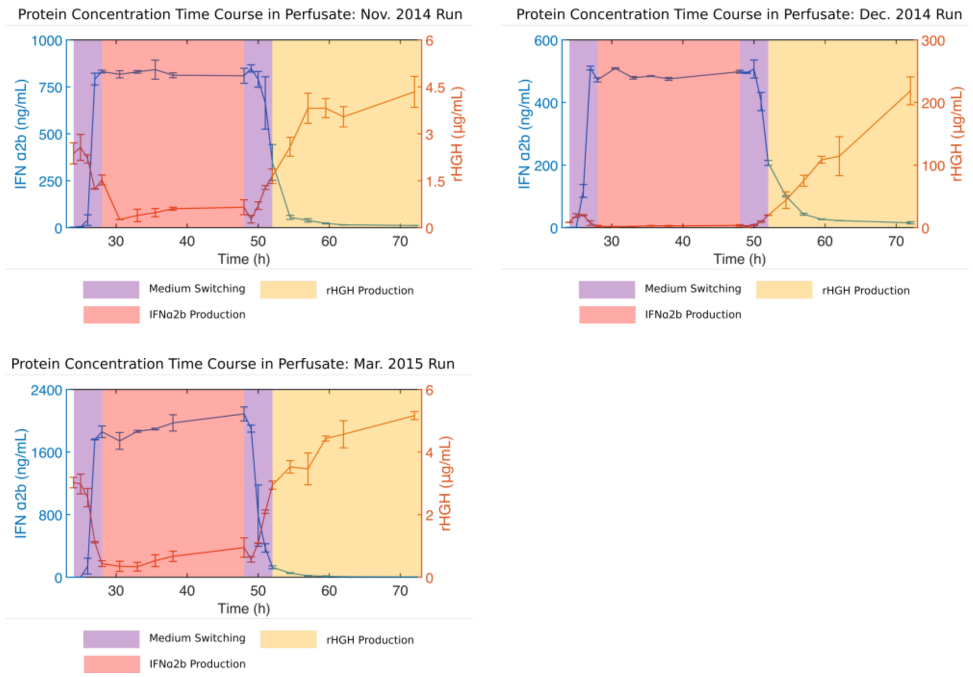

Supplementary Figure 10: Protein production time course for three additional microbioreactor runs. Each run consists of two independent microbioreactors operating the same protocol presented in the manuscript in parallel. The cumulative protein production quantity is summarized in Supplementary Table 4.

Name	RefSeq	Size (Mb)	GC%	Genes
Chr 1	NC_012963.1	2.8	41.0	1,538
Chr 2	NC_012964.1	2.39	41.0	1,333
Chr 3	NC_012965.1	2.25	41.1	1,198
Chr 4	NC_012966.1	1.78	41.4	971

Supplementary Table 1: DNA content of chromosomal DNA in *Pichia pastoris*.

Integration Site	Chromosome	Begin	End
1	2	286,540	286,072
2	1	8,346	9,028
3	1	1,386,085	1,386,686
4	2	493,919	494,400
5	3	292,747	293,351
6	3	1,156,771	1,157,374
7	4	1,547,467	1,547,086
8	2	808,602	809,080
9	2	286,989	286,140

Supplementary Table 2: Chromosomal locations of homology regions used in the vectors that were used to integrate landing pads in the genomic DNA of *Pichia Pastoris*.

Figure	Notes	Plasmid Name	Addgene #
Figure 2	P: scTEF1, mP: GAP	PP255	78934
Figure 2	BxBI expression	PP43	78953
Figure 2	R4 expression	PP44	78954
Figure 2	TP901-1 expression	PP45	78955
Figure 3	Strain 1	PP295	78935
Figure 3	Strain 2	PP296	78936
Figure 3	Strain 3	PP297	78937
Figure 3	Strain 4	PP298	78938
Figure 3	Strain 6	PP299	78939
Figure 3	Strain 7	PP300	78940
Figure 3	Strain 5	PP318	78941
Figure 3	Strain 8	PP228	78942
Figure 3	Strain 9	PP322	78943
Figure 4	P: scTEF1, mP: mCYC	PP259	78961
Figure 4	P: GAP6, mP: mCYC	PP258	78962
Figure 4	P: ppTEF1, mP: mCYC	PP228	78942
Figure 4	P: GAP, mP: mCYC	PP257	78963
Figure 4	P: scTEF1, mP: AOX1	PP247	78964
Figure 4	P: GAP, mP: AOX1	PP245	78965
Figure 4	P: GAP6, mP: AOX1	PP246	78966
Figure 4	P: scTEF1, mP: GCW14	PP277	78967
Figure 4	P: GAP, mP: GCW14	PP275	78968
Figure 4	P: ppTEF1, mP: AOX1	PP190	78969
Figure 4	P: GAP6, mP: GCW14	PP276	78970
Figure 4	P: ppTEF1, mP: GCW14	PP273	78971

Figure 4	P: scTEF1, mP: GAP6	PP263	78972
Figure 4	P: GAP, mP: GAP6	PP261	78973
Figure 4	P: GAP6, mP: GAP6	PP262	78974
Figure 4	P: GAP6, mP: GAP	PP254	78975
Figure 4	P: scTEF1, mP: GAP	PP255	78934
Figure 4	P: GAP, mP: GAP	PP253	78976
Figure 4	P: ppTEF1, mP: GAP6	PP229	78977
Figure 4	P: ppTEF1, mP: GAP	PP244	78978
Figure 5	245R P: GAP, mP: AOX1	PP310	78979
Figure 5	246R P: GAP6, mP: AOX1	PP311	78980
Figure 5	255R P: scTEF1, mP: GAP	PP362	78981
Figure 5	246B P: GAP6, mP: AOX1	PP324	78982
Figure 5	245B P: GAP, mP: AOX1	PP326	78983
Figure 5	255B P: scTEF1, mP: GAP	PP363	78984
Supp 3	Integration Site 1	PP74	78944
Supp 3	Integration Site 2	PP75	78945
Supp 3	Integration Site 3	PP76	78946
Supp 3	Integration Site 4	PP77	78947
Supp 3	Integration Site 5	PP78	78948
Supp 3	Integration Site 6	PP79	78949
Supp 3	Integration Site 7	PP69	78950
Supp 3	Integration Site 8	PP67	78951
Supp 3	Integration Site 9	PP151	78952
Supp 4	GCW14	PP165	78985
Supp 4	Long ppTEF1	PP152	78986

Supp 4	scTEF1	PP87	78987
Supp 4	Short ppTEF1	PP164	78988
Supp 4	WT GAP	PP149	78989
Supp 4	No promoter	PP093	78990
Supp 4	GAP1	PP154	78991
Supp 4	GAP2	PP160	78992
Supp 4	GAP3	PP161	78993
Supp 4	GAP4	PP155	78994
Supp 4	GAP5	PP162	78995
Supp 4	GAP6	PP163	78996
Supp 4	GAP7	PP153	78997

Supplementary Table 3: List of constructs used in this manuscript.

	IFN α2b Production (20 hours)	rHGH Production (20 hours)	IFN α2b Leakage (20 hours)	rHGH Leakage (20 hours)	Wet Cell Weight
Nov. 2014 Run	8.17±0.31 μg	38.9±4.2 μg	0.223±0.033 μg	5.45±1.66 μg	387±44 g/L
Dec. 2014 Run	4.93±0.04 μg	1.52±0.18 mg	0.319±0.026 μg	0.03±0.0079 mg	349±8 g/L
Mar. 2015 Run	19.78±0.74 μg	45.8±2.2 μg	0.137±0.026 μg	7.09±2.36 μg	351±26 g/L

Supplementary Table 4: Production summary for IFNα2b and rHGH and wet cell weight measurement for the three additional microbioreactor runs in Supplementary Figure 10. All data are averaged across two independent microbioreactors running the same protocol in parallel. Values represent mean and s.e.m. (n=2).
